# 

**Published:** 1872-03

**Authors:** 


### CONTENTS.

COMMUNICATIONS.

Running the Risk ........ 97
Filling Teeth ........ 106
Office Notes—Destroying Nerves ........ 115
Neuralgia ........ 118
Two Useful Things ........ 120

PROCEEDINGS OF SOCIETIES.

Discussion before the Sixth Annual Meeting of the Ohio State Dental Society, December 5-6, 1871 ........ 122
Eastern Ohio Dental Association ........ 133

EDITORIAL.

Ohio Dental College ........ 136
Mississippi Valley Dental Society Meeting ........ 137
Ohio College of Dental Surgery ........ 138
Mass Convention of the Dentists of the West ........ 139

